# The Role of Lymph Node Dissection in the Treatment of Bladder Cancer

**DOI:** 10.3389/fsurg.2018.00062

**Published:** 2018-10-05

**Authors:** Francesco Cattaneo, Giovanni Motterle, Filiberto Zattoni, Alessandro Morlacco, Fabrizio Dal Moro

**Affiliations:** ^1^Clinica Urologica, Dipartimento di Scienze Chirurgiche, Oncologiche e Gastroenterologiche, Università degli Studi di Padova, Padova, Italy; ^2^Clínica Urologica, Ospedale “Santa Maria della Misericordia”, Università di Udine, Udine, Italy

**Keywords:** bladder cancer, lymph nodes, lymph node dissection, radical cystectomy, nodal disease

## Abstract

Lymph node dissection (LND; PLND: pelvic LND) is an essential component of radical cystectomy (RC) for bladder cancer (BC). However, the optimal anatomical extent of LND and its potential therapeutic role are still controversial: as we will explain, the extent of LND dissection is a predictor of survival and local recurrence but what is an adequate extension is still unclear. Moreover, there is large uncertainty about the role of surgery in patients with clinically-positive nodes. In this review we will provide a synthesis of the available evidence on this highly debated topic. Overall, the studies presented in this work support the idea that extended lymphadenectomy could provide optimal diagnostic and possibly therapeutic results in cN- patients. In cN+ patients, post chemotherapy surgery may be considered especially in subjects who have a good response to CHT, although definitive evidence is still needed. Finally, the final results of randomized trials are eagerly awaited to draw definitive conclusions of the role of PLND in BC.

## Introduction

Radical cystectomy (RC) plus regional lymph node dissection (LND) is the gold standard in the treatment of high-risk non-muscle-invasive bladder cancer (NMIBC) unresponsive to intravesical therapies or muscle-invasive bladder cancer (MIBC). Neoadjuvant chemotherapy is recommended as part of the treatment (for cT2-T4a N0 MIBC. RC includes the removal of the bladder itself and its surrounding perivescical fat. In men RC consists in removing also the prostate and seminal vesicles whereas in woman it includes the ovaries, uterus with cervix, and anterior vagina (1).

LND is an essential step in the treatment of MIBC because it is known that approximately 25–30% of patients will have lymph node metastasis at the time of surgery (2–4) and also because lymph node status is one of the most important indicators of long term overall survival (OS) and recurrence-free survival (RFS) (5).

As we will analyze more in detail in this paper, the extent of LND dissection is a predictor of survival and local free recurrence but what is an adequate extension is still unclear.

The rationale of removing any positive lymph node appears obvious because it provides a more complete removal of cancer and a better stratification and staging of the patient for further adjuvant therapies; however, the evidence supporting this approach is limited in quality, quantity, and somewhat controversial. According to the known pathophysiology of BC metastases, LND might be a helpful step also in pN0 patients because it could help remove immunosuppressive factors that could to facilitate distant metastases.

## Anatomical perspective of lymph node dissection for bladder cancer

**Figure 1:**
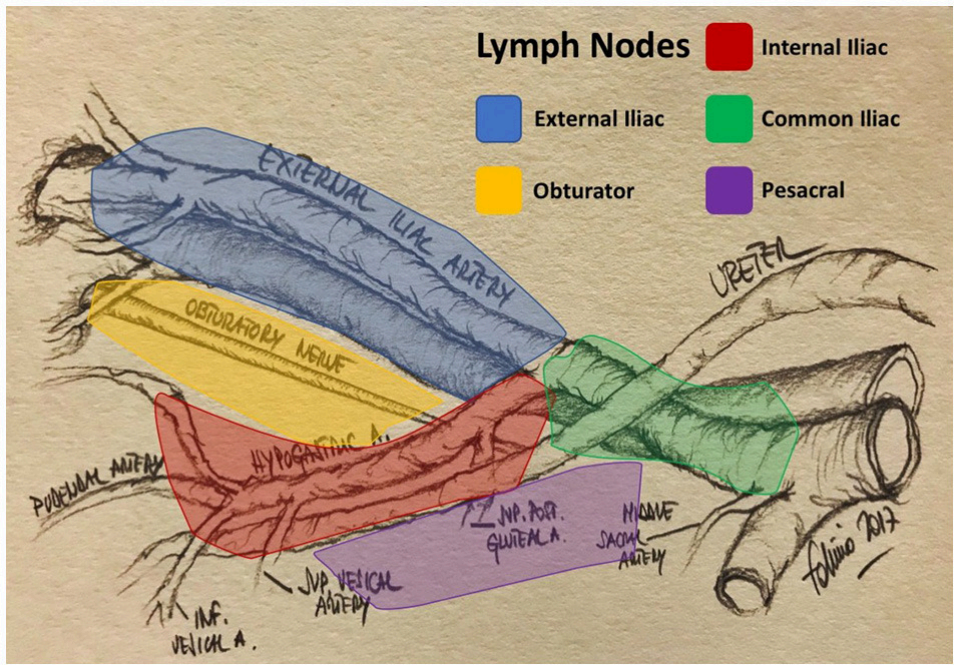
Pelvic lymph node areas (original drawing by the author).

**Figure 2:**
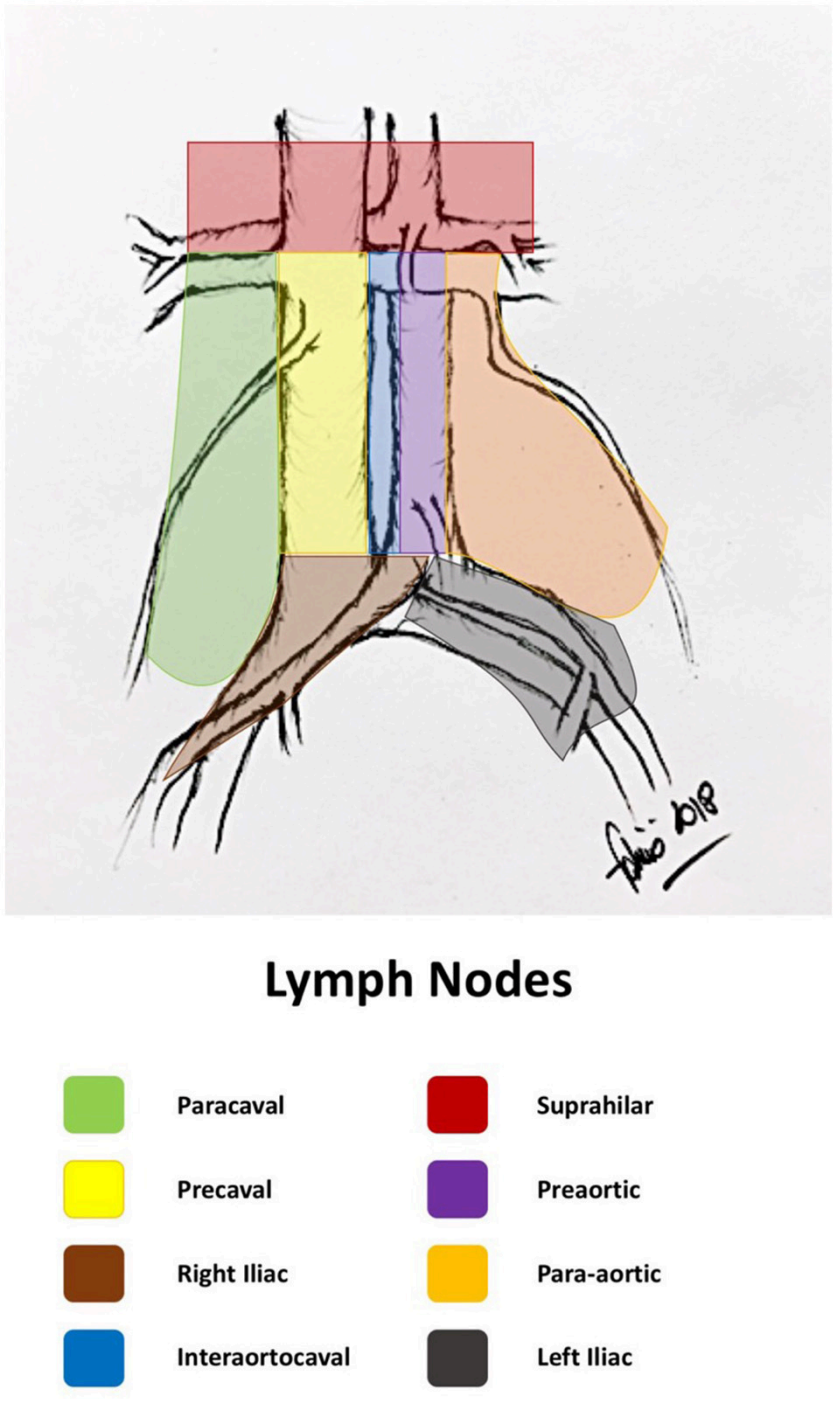
Retroperitoneal lymph node areas (original drawing by the author).

The primary lymphatic drainage site for bladder cancer includes the internal iliac, external iliac, obturator, and presacral lymph nodes. Secondary drainage sites include the common iliac, para-aortic, interaortocaval, and paracaval lymph nodes (Figures 1, 2) (6, 7).

Although “skip” metastasis (meaning metastases in secondary drainage sites without evidence of metastases in primary sites) appears to be a relatively rare event in bladder cancer, it has been reported in the literature. Leissner et al. in their series found that 6.9% of patients had nodal metastasis in lymph nodes above the common iliac bifurcation but none above the aortic bifurcation; therefore, to achieve an accurate LN staging, it would be necessary to dissect up to the aortic bifurcation. (8).

Tarin et al. (9) evaluated 591 RC patients. LN involvement was identified in 114 patients (19%). Stratifying by tumor stage <pT2, pT2, pT3, and pT4, LN involvement was identified in 6, 18, 40, and 12 patients 60%, respectively. In this group, seven patients (6%) had no positive lymph nodes within the true pelvis (skip lesions). Since skip lesions are known to be very rare, this phenomenon may be the result of missed positive LNs in the true pelvis or of a specimen-labeling error.

Also mapping studies have found that a very low number of patients had metastases in regions above the bifurcation of aorta without synchronous metastases in the true pelvis. These observations strengthens the idea that “skip” metastasis is an exception rather than the rule (9).

In a recent series Moschini et al. evaluated 653 patients with cN0cM0 high risk NMIBC or MIBC treated with RC and extended or super-extended PLND without neoadjuvant CHT. 29.3% of patients had pathologically confirmed node metastasis. Most patients were found with node metastases within the standard template (26.3%), on the other hand 4.6% and 3.2% patients had node metastases in extended and super-extended templates, respectively. However, of these only 2 patients were found without concomitant lymph node metastases in the limited or standard templates (3). According to this study, superextended LND template might be superfluous in the large majority of patients for staging alone, since most patients with positive nodes in the extended or superextended templates will have positive nodes also in the limited or standard fields.

## Patterns of nodal spread in muscle-invasive bladder cancer

One autopsy study evaluated the metastatic behavior of bladder tumors and showed that, in 251 of 367 patients (68%) with metastatic MIBC, the most frequent sites of metastases were: regional lymph nodes (90%: 92% in perivesical or pelvic, 72% in retroperitoneal, and 35% in abdominal lymph nodes), liver (47%), lung (45%), bone (32%), peritoneum (19%), pleura (16%), kidney (14%), adrenal gland (14%), and the intestine (13%). The frequency of metastases increased with local tumor extension and there was a strong association between the presence of lymph node and distant metastases (47% of the patients had both). This association suggests that distant metastases could mostly be derived from the regional metastases and as a consequence lymphadenectomy could theoretically improve the prognosis (10).

LND plays an important role also in high risk NMIBC undergoing RC as we can see from Bruins' work where of 114 patients who underwent RC for NMIBC, 9 % with cT1, 12 % with cTis, and 0% with cTa had lymph node metastasis (4).

## More is better? extent of LND

Proven the importance of LND as part of staging and treatment of BC, there are still some questions that need to be answered. Does the extent of LND give a survival benefit? And which is more important between the anatomic extent of dissection and the number of nodes removed?

The anatomic extent of PLND necessary for loco-regional disease control and reliable staging is controversial, and currently no dissection template has been universally accepted. Furthermore, there has been no uniformity in reporting and measuring the dissection extent; thus, the most commonly used measure of the extent of PLND has been the lymph node count.

However, although descriptions of the anatomic extent of lymphadenectomy somewhat differs among the published studies, the extent of LND was determined a priori based on discussion in an expert panel (EAU Working Group on MIBC) and was categorized as follows:

- Limited is defined as the removal of obturator and internal iliac nodes,
- Standard included also the external iliac nodes,
- Extended included also common and presacral nodes,
- Super-extended included all the nodes removed along the inferior mesenteric artery (11).

It has been shown that limited PLND removes only about 50% of all primary lymphatic landing sites. In order to remove 90%, PLND should be extended to include LNs lateral and medial to the internal iliac vessels, and the common iliac region up to the uretero-iliac crossing (12).

Several factors might influence lymph node count such as the method of lymph node submission (en-bloc vs. separate packets and the number of packets sent), surgical technique, and variability in the pathologic practices and reporting standards. Last but not least, there is an important inter-individual variability in the number of lymph nodes that can be retrieved from the same template (13).

Reports in the literature regarding the correlation between the number of dissected nodes and the prognosis following RC are conflicting. Li et al. performed a meta-analysis of 41.400 patient who underwent RC, of which 6.044 were pN+. In their study they showed that a greater extent of LND during RC had statistically significant advantages in terms of OS, CSS and RFS, corresponding to reduced risks of 28, 34 and 36%, respectively, compared with patients with a lesser extent of LND (14).

The number of resected nodes showed positive correlation also regarding local recurrence rates (*p* = *0.002* in > 11 nodes in pN+ patients) (15) and a stronger association with survival (HR 2.0, *p* = 0.001) (16).

Cole et al. used the SEER data to analyze adequacy of LND (defined by >10 nodes removed) during years from 1988 to 2010 and found that, in the total sample, only 45% of patients received and adequate LND, with a proportion increasing over time from 26.4 to 61.3% (17).

Dhar and al. found that 5-years RFS was 23 vs. 57% (*p* < 0.0001), and OS was 26 vs. 46 % (*p* = 0.0021), in favor of the extended LND group compared to limited LND group. For pN+ patients the 5-year relapse-free survival and overall survival were both 7% for a limited dissection compared with 35 and 34% for patients undergoing extended LND, respectively (*p* < 0.0001) (18).

A super-extended dissection (up to the inferior mesenteric artery) resulted in higher node count (median 38 vs. 22, *p* < 0.0001) without survival benefit (19). This lack of survival advantage was confirmed in another study from Bruins (11).

Other retrospective series examined the relation between the extent of lymph node dissection, as defined by the number of lymph nodes removed, and survival in patients with or without lymph node metastases (20). Data supported the role of extended PLND in improving survival in both node positive and node negative BCa patients (18, 20). Considering demographics and pathological features, clinical T and N stage were predictors of the possibility to harbor node metastases in the extended or super-extended template (3).

In a meta-analysis of all studies comparing extended and standard PLND, overall odds ratio of 5-year recurrence-free survival rate was 1.63 (95% CI 1.28-2.07, *p* < 0.001), suggesting a significant benefit for the extended PLND with no increase in mortality and/or morbidity (21). In a recent systematic review, the influence of LND on perioperative and oncologic outcomes in patients undergoing RC for MIBC was assessed including 23 studies reporting on 19.793 patients (11). Of interest, in this last study, the meta-analysis originally planned by the authors was not possible, due to the large heterogeneity between studies. However, the final results suggested that in terms of oncologic outcomes, LND of any extent is better than no LND; furthermore, extended LND might improve oncologic outcomes compared with more limited types of dissection, although extending the dissection beyond the boundaries of eLND (i.e., super-extended LND) is unlikely to lead to any further benefits. Despite the evidence summarized in this review was not strong enough to provide firm recommendations regarding the most optimal extent of LND, the included studies fairly consistently report an oncologic benefit for eLND compared with less extended LND templates.

On the other hand, preliminary available prospective evidence did not prove a survival benefit of extended PLND compared to limited PLND. The phase 3 trial conducted of Association of Urogenital Oncology and German Cancer Association (LEA trial) examined recurrence free survival (primary endpoint) and cancer-specific survival (secondary endpoint) of extended PLND (including 14 fields, up to the inferior mesenteric artery) vs. limited PLND (including 6 fields: bilateral obturator, internal and external iliac nodes). There was only a trend but no significant difference in terms of improved RFS and CSS with an extended PLND. On a *post-hoc* analyses, a survival benefit was seen only for patients who harbored an organ confined disease (22).

Another randomized clinical trial of the National Cancer Institute (SWOG 1011), active in USA and Canada, is still in progress, with the primary aim of examining disease free survival of extended (including common iliac and presacral PLND) vs. standard PLND (23). The estimated end of this study is 2022 and the planned enrollment 620 participants.

Therefore, further data from on-going randomized clinical trials on the therapeutic impact of the different extents of lymphadenectomy are awaited.

The diagnostic performances and the survival outcomes of the articles presented in the text are summarized in Table 1.

**Table 1:** Diagnostic and survival outcomes of different LND approaches.

**Authors**	**Percentage of postive nodes (%)**				**Nodes removed (mean)**			**Survival benefit**				
	**Not specified**	**Standard**	**Extended**	**Super–extended**	**Standard**	**Extended**	**Super–Extended**	**No LND**	**Limited LND**	**Standard LND**	**Extended LND**	**Super–extended LND**
Tarin et al. (9)			19									
Moschini et al. (3)		26.3	4.6	3.2	20							
Li et at. (14)	14.6											
Herr et al. (16)	21							33%^*^	46%^*^	60%^*^		
Dhar et al. (18)		13	26		1	2				7%^**^	35%^**^	
Zehnder et al. (19)			28	35		22	38				54^***^	50^***^

* *5 years survival rate*.

** *5 years recurrence free survival*.

*** *5 years overall survival*.

## Treatment of node-positive patients

Clinically-positive lymph nodes (cN+) have usually been defined as pelvic nodes >8 mm or abdominal nodes >10 mm in maximum short-axis diameter as detected via preoperative computed tomography (CT) or magnetic resonance imaging (MRI). CT/MRI had limited ability in predicting pN+, mainly because of their inability to localize small volume, micro metastatic nodal disease. A multi-institutional study confirmed the poor accuracy of conventional preoperative imaging in assessing nodal disease status: cross-sectional imaging showed sensitivity of 18% and specificity of 96% for prediction of lymph node metastases, with accuracy of 78%. Therefore, the pathologic node status was the only reliable predictor of long-term outcome (poor survival), while cN+ status did not show an independent role as a predictor of oncologic outcomes and should be considered carefully before precluding potentially curative treatments (24).

A preoperative imaging method that accurately demonstrates the extent of involvement and therefore may guide the extent of surgical dissection could be desirable for both staging and cure purposes.

Although F-18 FDG PET and PET/CT are now commonly used in imaging of various cancers, their use in BC staging is limited by the high urinary excretion activity in the bladder and ureters. A recent meta-analysis showed a low sensitivity (0.57) and high specificity (0.92) for the detection of metastatic LNs in patients with newly diagnosed BC (25). (Tumour Biol. 2015 May;36(5):3209-14. doi: 10.1007/s13277-014-2361-7. Epub 2015 Mar 26. Diagnostic value of [18F] FDG-PET and PET/CT in urinary bladder cancer: a meta-analysis. Zhang H1, Xing W, Kang Q, Chen C, Wang L, Lu J.)—INSERIRE

Patients with clinically positive LN disease are generally considered for systemic platinum-based CHT in the induction setting and then RC, as consolidation, in those with a major response to the induction CHT (26, 27).

Neoadjuvant cisplatin-containing combination CHT for patients with muscle-invasive urothelial carcinoma (UC) has been reported to improve outcomes in several randomized trials (improved overall survival 5–8% at 5 years). However, most neoadjuvant trials have studied the effect of chemotherapy in patients with clinically negative nodes (cN0) and excluded patients with clinically node positive disease (cN1-3). Clinical LN metastases (cN+) are common in patients with advanced UC, and the prognosis of patients with cN+ is significantly worse than cN0. In the absence of visceral metastasis and despite CHT, the reported 5-yr overall (OS) rate was <20% (28).

LN status after CHT seems to be more important than local tumor status in evaluating survival in cN+ patients, as we can understand from Nieuwenzhuijzen work where a tumor negative bladder combined with tumor negative nodes were associated with improved survival (HR 4.4) as was a tumor negative LN region in the presence of residual bladder disease (HR 2.8) (29).

However, prognosis for cN+ BC remains poor despite the use of induction CHT and the use of CHT only probably represents an under treatment for most patients: an historical series from MSKCC showed that 92% of patients who did not undergo surgery after major response to CHT died of metastatic disease, while a third of patients who had complete response to CHT and surgery had long-term survival (30). However, CHT is a milestone in the management of these patients: more recently, Galsky et al. (31) evaluated a large number of patients with cN+ using the National Cancer Data Base and comparing the effects of CHT and/or RC (19). From their results, an multimodal approach integrating perioperative CHT was associated with better outcomes than RC alone. The 5-year OS for pre-operative CHT and RC, RC and adjuvant CHT, and RC alone were 31, 26, and 19%, respectively. Although the optimal sequence and modalities remain incompletely defined, data suggested a survival benefit with either CHT used before RC or in the adjuvant setting. Overall, the evidence collected suggests a benefit for RC after complete or major response to systemic CHT (32).

In Meijer series 1 of 4 patients showed complete pathologic response to induction CHT with subsequently a significant CSS benefit (median CSS 127 months and 5-year CSS 63.5%) (33). Several studies assessed pathological and survival outcomes in patients with cN1-3 disease treated with induction CHT and RC. Partial pathological response (pPR) was defined as down staging to non-muscle invasive disease, pT1N0 or less, and complete pathological response (pCR) was defined as pT0N0. In the largest series published, in which 304 patients received induction CHT, the pCR and pPR rates for the entire cohort were 14.5 and 27%, respectively (34). In the same work, pN0 status, number of LNs removed (>15), negative soft tissue surgical margins, and cisplatin-based CHT were independently associated with improvement in overall survival, while no difference was seen in survival outcomes between cN1 and cN2-3 patients and between the different chemotherapy regimens. However, a known limitation of this study is that patients who received CHT for cN+ disease but did not proceed to surgery were not included in the analysis, obviously creating a selection bias toward patients with good response to CHT and better prognosis.

High volume nodal disease is very unlikely to be missed by modern imaging techniques, but it is still possible to discover grossly enlarged LN at the time of surgery for RC. This could be in part due to the latency between the initial imaging staging and the date of surgery, with can be significant especially in some systems (35). Even in this context, the available evidence suggests that RC with extensive LND should not be discontinued. As shown by Herr et al in the pre-CHT era (36), a non-negligible proportion of patients (24%) could be cured with RC and eLND, especially those in whom primary tumor is clinically confined to the bladder (stage T2). On the other hand, results from studies evaluating the outcome when surgery was aborted due to gross LN involvement and/or extensive extravesical extension revealed poor outcomes (37).

## Conclusion

Bilateral pelvic lymphadenectomy is an important part of RC for BCa. Lymphadenectomy, completed according to the extended template, provides optimal diagnostic and possibly therapeutic results. The final results of two randomized trials (LEA and SWOG S1011) are anxiously awaited to define finally the appropriate extent of PLND. Post chemotherapy surgery may be used in patients with clinically evident pelvic or even retroperitoneal lymph nodal metastases, especially if they have a response to CHT, although definitive evidence is still needed.

## Author contributions

FC, GM, FZ, AM, and FD made a substantial contribution to this article in terms of conceiving, writing, and reviewing the manuscript.

### Conflict of interest statement

The authors declare that the research was conducted in the absence of any commercial or financial relationships that could be construed as a potential conflict of interest.
